# Gelastic Seizures in a Patient with Right Gyrus Cinguli Astrocytoma

**Published:** 2010-11-25

**Authors:** L Nicolae, G Iacob, M Poparda, BO Popescu

**Affiliations:** * Department of Neurosurgery, University Hospital BucharestRomania; ** Department of Neuropathology, University Hospital BucharestRomania; *** Department of Neurology, University Hospital BucharestRomania; **** Laboratory of Molecular Medicine, ‘Victor Babes’ National Institute of Pathology, BucharestFrance

**Keywords:** gelastic seizures (GS), dacrystic seizures (DS), epilepsy surgery, gyrus cinguli, cerebral astrocytoma

## Abstract

**Objective and importance**: Gelastic seizure (GS) also known as ‘gelastic epilepsy’ is a rare type of seizure associated with several different conditions such as tumors–hypothalamic hamartromas, tuberous sclerosis, hemangiomas, post infectious foci, cortical temporal dysplasia. We report one case of this rare condition generated by a right gyrus cinguli gr. Ⅱ astrocytoma.

**Clinical presentation**: A 27 years old, male, right handed, was admitted for a 2 years history of very frequent gelastic seizures accompanied sometimes by simple motor partial seizures in both arms, more often being involved his left arm, without impairment of his consciousness state. His neurological examination was normal. Diagnosis was made on native CT scan: minimal hypodense frontal right paramedian lesion, cerebral MRI showed a small right, parenchymal,  homogeneous  lesion (16/22/15mm), well delimited, involving gyrus cinguli, without perilesional edema and mass effect, hyperintense both on T_1_ and T_2_ MR sequences, non–enhancing after Gadolinium. The cerebral lesion was also documented on  EEG and video–EEG recordings. Using an interhemispheric microsurgical approach, above the corpus callosum and the right pericallosal artery, at the level of gyrus cinguli, a yellow–gray, infiltrative tumour, having a moderate vascularisation, has been identified and totally removed. The anatomo–pathological analysis revealed a grade Ⅱ astrocytoma. The patient recovered very well, without deficits, no gelastic seizures or epileptic manifestations; three months after operation he is still free of seizures.

**Conclusion**: A case of gelastic seizures accompanied by simple motor partial seizures in both arms, without impairment of his consciousness state induced by a gradeⅡright gyrus cinguli astrocytoma is described and documented by radiological and electrophysiological studies. Using microsurgical resection, the tumor was totally removed, the patient clinical condition improved. Without an affective connotation as in temporal or hypothalamus topography, gelastic seizures are not patognomonic for hypothalamic hamartomas and in the case of frontal localization of the lesion they can be associated with motor involvement of the limbs as in our case.

## Introduction

GS a rare form of epilepsy, involves sudden outburst of laughter or crying with no apparent cause, the laughter may sound unpleasant, sardonic rather than joyful, usually lasts for less than a minute [1]. Conscious state may not be impaired, although this is often difficult to asses particularly in young children. After a seizure it could be seen: tonic–clonic, atonic seizures, strange eye movements, lipsmacking, fidgeting, mumbling, twitching, bursts of cooing, giggling, smiling causing learning disabilities, faulted cognitive function, also flushing, tachycardia, altered respiration are widely recognized. GS can be cryptogenic or symptomatic for a variety of cerebral lesions, usually manifests at children with hypothalamic hamartromas [2].

## Case Report

A 27 years old, male, right handed, was admitted for a 2 years history of very frequent gelastic seizures. The patient described aura–like symptoms: especially warmth throughout the face and dizziness that preceded the seizure and he denied any affective connotation. Some of the seizures consisted only of a brief period of laughter without any motor involvement, but a typical seizure would consist of sudden laughter accompanied by simple motor partial seizures in both arms, more often being involved his left arm, without impairment of his consciousness state, which lasted less than 1 minute. He had no previous past medical or social history other than being a smoker: 10–15 cigarettes per day. The frequency of the seizures was initially low, but increased progressively over time to about 8–10 seizures/day, despite anti–epileptic treatment – at the time of admission he was taking Keppra – Levetiracetam 3 x 250 mg/day and Phenytoin 3 x 100 mg/day. With the Neurology Electrophysiology Department assistance we have been able to record a couple of seizures on the Video–EEG His neurological examination was normal.

**Figure 1:**
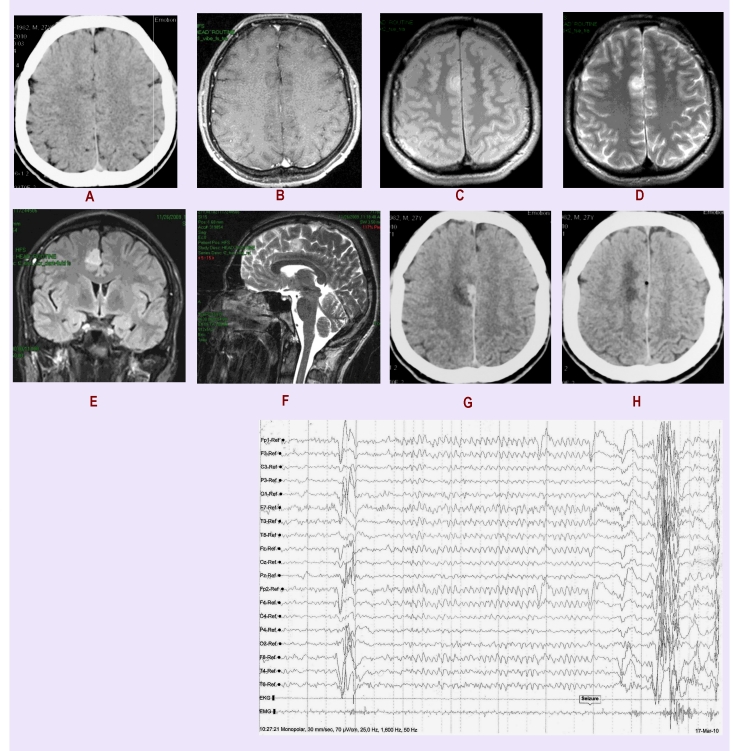
A. Preoperatory native CT scan: minimal hypodense frontal right paramedian lesion, B. T_1_ cerebral MRI small hyperintense parenchymal  homogeneous  lesion 16/22/15mm, well delimited, involving gyrus cinguli, without perilesional edema and mass effect, C hyperintense lesion on T_2_ MR sequences, non–enhancing after Gadolinium, D coronal lesion T_1_ MR sequences, E sagital lesion T_2_ MR sequences, G and H Postoperatory CT scan, I EEG recording: medio–frontal origin and rare interictal epileptiforme elements

Diagnosis was made on native CT scan: minimal hypodense frontal right paramedian lesion (Figure 1A), cerebral MRI (Figure 1 B–F) showed a small right parenchymal  homogeneous  lesion 16/22/15mm, well delimited, involving gyrus cinguli, without perilesional edema and mass effect, hyperintense both on T_1_ and T_2_ MR sequences, non–enhancing after Gadolinium. The cerebral lesion was also documented on  EEG and video–EEG recordings: 3 seizures with medio–frontal origin and rare interictal epileptiform elements were recorded (Figure 1I)

Using an interhemispheric microsurgical approach, above the corpus callosum and the right pericallosal artery, at the level of gyrus cinguli, a yellow–gray, infiltrative tumour, having a moderate vascularisation had been identified and totally removed. The anatomo–pathological analysis revealed a grade Ⅱ astrocytoma. The patient recovered very well, without deficits, no gelastic seizures or epileptic manifestations; three months after operation he is still free of seizures. A control postop CT revealed no tumor (1 G, H)

## Discussions

The term ‘gelastic’ comes from the Greek word ‘gelos’ which means laughter to emphasize the main characteristic of these seizures [1][2]. Trousseau 1877 [3] was the first to describe laughing seizures to a patient with vertigo, jerking bursts of laughter using the term ‘epileptic vertigo’. Gowers 1881–cited by [1] observed emotions with a cheerful character as a part of a seizure. Daly and Mulder 1957 [4] coined the term ‘gelastic epilepsy’. Gascon and Lombroso 1971 [5] suggested the importance of diencephalic lesions in many cases as a cause of the GS; also defined criteria for such condition: stereotyped recurrence of bouts of laughter in absence of external precipitants, concomitant manifestations accepted as epileptic, presence of interictal electroencephalography abnormalities and absence of conditions that can cause pathologic laughter. Pendl 1975–cited by [1] facial contraction in the form of a smile are usually combined by laughter–like vocalization. Penfold 1978 , Tassinari 1994–cited by [1] GS could be mistaken by normal laughter or misdiagnosed as infantile colic. Cerullo 1998–cited by [1] identified GS associated with autonomic features such as tachycardia, flushing, altered respiration; mirth sensation is not frequent, also conscious state may not be impaired, (difficult to asses in young children). Striano 1999–cited by [1] described unpleasant epigastric sensation. Sturm 2000–cited by [1] described an urge to laugh to some patients that sometimes can be suppressed. Sethi and Rao 1976 [6] some patients can experience both gelastic and crying seizures which are termed ‘dacrystic’–DS or ‘quiritarian’ seizures: clusters at sleep or moan at the onset, with face–flushing that rapidly evolves into crying, associated with facial oro–alimentary automatisms Lopez–Laso 2007–cited by [1].

GS have been associated classically to hypothalamic hamartomas–congenital benign mass of glial tissue on or near the hypothalamus presenting with the classic triad of gelastic epilepsy, precocious puberty–secondary sex characteristics before the age of eight at girls due to early hypothalamic pituitary gonadal axisactivation, developmental delay [7]. GS were also described in several different conditions: brain tumors [1], frontal [8–11], temporal [12], parietal [13] lobe lesions, dilated temporal horns, atrophy, tuberous sclerosis, post infectious foci, cortical dysplasia [14]. GS originated over the anterior cingulate region was reported by McConachie and King 1997 [15], Mohamed–2007 [16]. GS can be detected with EEG–ictal and interictal discharges, SPECT–single photon emission computed tomography, MEG–magnetoencephalography, but imagistic investigations should always be obtained to try to localize a possible brain lesion responsible for the epilepsy such as computed tomography, magnetic resonance imaging, cerebral angiography [1]. Therapy options [17–20] are depending solely on the individual patient's pathology: surgery–if the seizures are caused by a tumor, Gamma knife for residual lesions, hormonal treatment can be attempted to help individuals with precocious puberty, while antiepileptic drugs are generally not responsive.

Differential diagnosis is an important part in the diagnostic process and it has to be made both clinical (‘forced laughter’ is well recognized a a symptom of pseudo–bulbar palsy in motor neurone disease or as an effect of bilateral pyramidal damage from cerebrovascular disease; it may also be occasionally observed in disseminated sclerosis) and imagistic–for example to differentiate between tumoral lesions and cortical displasias, in the later case imaging should show abnormalities that extend from cortex to germinal zone near the ventricular margin [21].	

## Conclusions

GS are not patognomonic for hypothalamic hamartomas and in the case of frontal localization of the lesion they can be associated with motor involvement of the limbs. They don't have an affective connotation, unlike the cases with temporal or hypothalamic focus. Thoroughly investigating the patient both by imagistic and electrophysiological means is mandatory for reaching a diagnosis and to be able to make a treatment plan, because GS generally are deteriorating into more complex seizure disorder resulting in intractable epilepsy. The main goal of treatment is to render the patient free of seizures so that he can have a normal social life and in case of tumors to prevent their advance, recurrence and possible future neurological deficits. When surgery is an option (for lesions like tumors or cortical dysplasias) most of these goals can be successfully attained.
